# Effects of an Aβ-antibody fragment on Aβ aggregation and astrocytic uptake are modulated by apolipoprotein E and J mimetic peptides

**DOI:** 10.1371/journal.pone.0188191

**Published:** 2017-11-20

**Authors:** Laia Montoliu-Gaya, Sandra D. Mulder, Robert Veerhuis, Sandra Villegas

**Affiliations:** 1 Departament de Bioquímica i Biologia Molecular, Facultat de Biociències, Universitat Autònoma de Barcelona, Bellaterra, Barcelona, Spain; 2 Clinical Chemistry Department, Amsterdam Neuroscience, VU University Medical Center, Amsterdam, The Netherlands; 3 Psychiatry Department, Amsterdam Neuroscience, VU University Medical Center, Amsterdam, The Netherlands; Weizmann Institute of Science, ISRAEL

## Abstract

Aβ-Immunotherapy has long been studied in the treatment of Alzheimer’s disease (AD), but not how other molecules involved in the disease can affect antibody performance. We previously designed an antibody fragment, scFv-h3D6, and showed that it precludes Aβ-induced cytotoxicity by withdrawing Aβ oligomers from the amyloid pathway towards a non-toxic, worm-like pathway. ScFv-h3D6 was effective at the behavioral, cellular, and molecular levels in the 3xTg-AD mouse model. Because scFv-h3D6 treatment restored apolipoprotein E (apoE) and J (apoJ) concentrations to non-pathological values, and Aβ internalization by glial cells was found to be decreased in the presence of these apolipoproteins, we now aimed to test the influence of scFv-h3D6 on Aβ aggregation and cellular uptake by primary human astrocytes in the presence of therapeutic apoE and apoJ mimetic peptides (MPs). Firstly, we demonstrated by CD and FTIR that the molecules used in this work were well folded. Next, interactions between apoE or apoJ-MP, scFv-h3D6 and Aβ were studied by CD. The conformational change induced by the interaction of Aβ with apoE-MP was much bigger than the induced with apoJ-MP, in line with the observed formation of protective worm-like fibrils by the scFv-h3D6/Aβ complex in the presence of apoJ-MP, but not of apoE-MP. ScFv-h3D6, apoJ-MP, and apoE-MP to a different extent reduced Aβ uptake by astrocytes, and apoE-MP partially interfered with the dramatic reduction by scFv-h3D6 while apoJ-MP had no effect on scFv-h3D6 action. As sustained Aβ uptake by astrocytes may impair their normal functions, and ultimately neuronal viability, this work shows another beneficence of scFv-h3D6 treatment, which is not further improved by the use of apoE or apoJ mimetic peptides.

## Introduction

Alzheimer's disease (AD) is a neurodegenerative disorder characterized by a progressive decline in cognitive functions. According to the amyloid cascade hypothesis, the initial seed that initiates the disease progression is the accumulation of the amyloid-β (Aβ) peptide[1]. This can result from an increase in its production, as in the case of familial AD (FAD), or by a decrease in its clearance, which is likely the case in sporadic, mostly late-onset AD (LOAD)[2,3]. Aggregation and accumulation of Aβ result in alterations in synaptic function, activation of glial cells, release of inflammatory mediators, and oxidative stress[4,5]. Eventually, this accumulation may lead to the deposition of amyloid plaques in the brain, one of the histological hallmarks of AD[6].

Both fibrillar and diffuse plaques include components that co-localize with Aβ-deposits[7] and modulate fibril formation[8], known as amyloid-associated proteins (AAPs). The best characterized of these proteins is apolipoprotein E (apoE), a key protein involved in lipid metabolism[9]. Human apoE is a 299-residue glycoprotein composed of two separate domains joined by a flexible hinge region: the N-terminal domain, which constitutes the receptor-binding region, and the C-terminal domain, the lipid-binding region[10]. Epitope mapping of the apoE-Aβ complex revealed that Aβ can interact with both the lipid-binding site and the receptor-binding site within apoE[11]. Human apoE exists in three isoforms, apoE2, apoE3 and apoE4, with apoE3 as the most common form and apoE4 being the major genetic risk factor for AD[12].

Genome wide association studies (GWAS) have also identified *CLU/APOJ* as a genetic determinant for LOAD[13,14]. Apolipoprotein J (apoJ, clusterin) is a multifunctional protein normally associated with lipids in plasma and cerebrospinal fluid (CSF), and secreted as lipoproteins by hepatocytes and astrocytes[15]. In conjunction with apoE and some other AAPs, apoJ has been found associated with parenchymal and vascular Aβ peptide deposits in AD, already in early stages when Aβ deposits are diffuse[7,16]. Furthermore, apoJ can form soluble complexes with Aβ which are readily detectable in the CSF[17].

In previous studies we observed that Aβ internalization by adult human glial cells was negatively affected by apoE and apoJ[18,19]. Astrocytes produce the majority of apoE and apoJ in the central nervous system (CNS)[20,21] and the presence of reactive astrocytes around Aβ plaques suggests this reactive phenotype may play an important role in AD pathogenesis[22,23]. Astrocytes are positioned between neurons and cerebral microvessels to translate information on the activity level and energy demands of neurons to the vascular cells in the blood brain barrier (BBB) and, in addition, they participate in the tri-partite synapse, where astrocytes communicate bidirectionally with neurons[24]. In contrast to neurons, which are highly vulnerable to Aβ exposure, astrocytes demonstrate relative resistance to Aβ toxicity[25]. However, as described by Sölvander *et al*., when astrocytes are overwhelmed with Aβ, their degradation system becomes ineffective and partly degraded Aβ is left, which may, instead of being beneficial promote spreading of AD pathology[26].

Because Aβ peptide accumulation may potentially generate neurotoxicity, one of the approaches that has emerged in the recent years as a promising treatment for AD is Aβ-immunotherapy. High affinity antibodies were developed to directly target the Aβ peptide and reduce its burden. Despite initial positive results, bapineuzumab, a humanized monoclonal Aβ N-terminal-specific antibody (mAb-3D6), failed in Phase 3 clinical trials due to dose-related severe adverse side-effects[27]. Because these side-effects have been linked to the activation of microglia by the Fc part of the mAb, single chain antibody fragments (scFv) lacking such a portion have been proposed as a safer therapeutic strategy. ScFv-h3D6 is a bapineuzumab derivative that has been demonstrated to preclude Aβ peptide-induced toxicity by withdrawing Aβ oligomers from the amyloid pathway towards the worm-like (WL), a non-toxic pathway featured by short and curved fibrils[28]. Furthermore, scFv-h3D6 has been proven to be effective at the behavioral, cellular and molecular levels in the triple transgenic mouse model for AD (3xTg-AD) harbouring *APP*_Swe_, *PS1*_M146V_, and *tau*_P301L_ transgenes[29,30]. The treatment improved cognition and reversed behavioral and psychological symptoms of dementia (BPSD)-like symptoms, protected from cell-death, decreased extracellular Aβ oligomers, and restored apolipoproteins E and J concentrations, whose levels are rather increased in the 3xTg-AD.

Even though Aβ-immunotherapy has long been studied in the treatment of AD, how other molecules involved in the disease could affect the antibody action remains elusive. Because obtaining full-length apolipoproteins for therapeutic use is an arduous procedure, we have combined scFv-h3D6 with apolipoproteins E and J mimetic peptides (MP) composed of essential structures within these apolipoproteins for binding to other molecules. The apoE mimetic peptide (MP) is composed of the receptor binding site sequence within apoE (residues 141–150, which are identical in all human apoE isoforms), linked to a Class A lipid associating domain similar to the lipid binding site of apoE[31]. The apoJ-MP corresponds to the 6^th^ predicted helix of the 17 potential G* amphipathic helices in the mature apoJ protein (residues 113 to 122), and is built by D-isomers to prevent degradation[32]. The apoE-MP, although initially intended to treat arthrosclerosis[33–35] is already reported in the literature to improve cognition, decrease amyloid plaque deposition and reduce the number of activated microglia and astrocytes in the APP/PS1ΔE9 mice[31]. The apoJ-MP, also intended to treat atherosclerosis[32], has been suggested as a therapy for inflammatory disorders other than atherosclerosis[36], but this is the first study assessing its effect in AD.

In this work, we have determined the effect of scFv-h3D6, apoE-MP and apoJ-MP, alone and combined, on Aβ aggregation and cellular uptake by human primary astrocytes. If and how interactions among them interfere with their individual effects, and on the formation of protective WL fibrils by the scFv-h3D6/Aβ complex, was studied by circular dichroism (CD), transmission electron microscopy (TEM) and indirectly by flow cytometry (with fluorescently labelled Aβ). Our results indicate that scFv-h3D6, apoE-MP and apoJ-MP prevent Aβ fibrillation, and at the same time, reduce Aβ oligomers uptake by human astrocytes. However, whereas their individual effects point in the same direction, preincubation together induces interactions that modulate Aβ uptake.

## Materials and methods

### ScFv-h3D6 expression and purification

ScFv-h3D6 expression was carried out using pET28a (+) vector and *Escherichia coli* BL21 strain (EMBL's bank, SV is an EMBL alumnus). Induction with 0.5mM IPTG (isopropyl *β*-D-thiogalactopyranoside) was performed at *D* = 0.7 and incubation in the shaker at 20°C for 18h. After three freeze-thaw cycles, the cellular pellet was sonicated for 10 min, at 70% duty cycle and output 9 (Sonifier 450, Branson). The protein was obtained by solubilizing the insoluble fraction in denaturing buffer (100 mM Tris-HCl, 10 mM GSH, pH 8.5, and 8M urea) and refolding by dilution (1:10) in ice-cold refolding buffer (100 mM Tris-HCl, 100 mM L-arginine and 0.15mM GSSG, pH 8.5) for 48h. Next, cationic exchange chromatography (Resource S6, GE Healthcare) using 5 mM Na_2_HPO_4_, pH 6.5, buffer and a gradient up to 15% buffered 1M NaCl was used to completely purify the protein, and in addition to separate the native state and the disulphide scrambled forms of scFv-h3D6. Finally, the protein was applied to Detoxi-Gel Endotoxin Removing columns (Thermo Scientific), to remove possible traces of lipopolysaccharides. The buffer was changed to PBS using PD-10 Desalting Columns (GE Healthcare), and protein aliquots (150 μL) were stored at −20°C until use. The molecular mass of the different batches of purified protein was confirmed by MALDI-TOF (matrix-assisted laser-desorption ionization-time-of-flight) spectrometry, and the protein concentration was determined from its absorbance at 280 nm using an absorbance coefficient of 1.06 absorbance-units·cm^-1^ for a 1 mg/mL native scFv-h3D6 [37].

### ApolipoproteinE and J mimetic peptides

The apoE mimetic peptide (MP) has the sequence: LRKLRKRLLRDWLKAFYDKVAEKLKEAF[31,33] and the apoJ-MP is formed by D-isomers of residues LVGRQLEEFL[32]. Both MPs were synthesized, protected at both termini and at >95% purity, by Caslo laboratories (Denmark).

### Aβ_1–42_ peptide

#### Aβ_1–42_ preparations for cell culture and FACS analysis

Aβ_1–42_ (Bachem) was dissolved in hexafluoro-isopropanol (HFIP) (Fluka) to obtain a monomeric Aβ_1–42_ solution. Next the solution was aliquoted, nitrogen flow dried and stored at -80°C until further use, as previously described[38]. For FACS analysis, separate fluorescent (FAM labelled) Aβ_1–42_ preparations enriched in oligomers or fibrils were prepared essentially as described before[18]. Briefly, 100μg aliquots of Aβ were resuspended, while vortexing, in DMSO that already contained (0.1mg/mL) FAM labelled Aβ (Anaspec). After sonication for 10 min, the sample with an Aβ concentration of 2.5 mM, was split in two. One was dissolved in phenol-red DMEM to 100 μM and incubated at 4°C for 24 hours to obtain oligomers. The other was dissolved to the same concentration in 10mM HCl and incubated at 37°C for 24h to obtain fibrils.

#### Aβ_1–42_ preparations for biophysical studies

The DMSO used to dissolve the Aβ for cell culture studies interferes with UV spectra. As previously described[39], Aβ peptide for biophysical experiments was resuspended in 20% (v:v) NH_4_OH, aliquoted and stored at -80°C. The Aβ in NH_4_OH was further diluted to 60 mM NaOH for aggregation and further diluted in either PBS (oligomers) or HCl (fibrils). Therefore, although proportions and procedures were the same as in Aβ for cell culture, no FAM-labelled Aβ or DMSO was used and NH_4_OH instead of HFIP was initially used to resuspend the peptide.

In both cases, and in order to assess the effect of scFv-h3D6, apoE-MP and apoJ-MP on Aβ oligomerization and fibrillation, the Aβ monomer preparation was allowed to form either Aβ oligomers (24h at 4°C) or Aβ fibrils (24h at 37°C) in the presence or absence of combinations of the different compounds.

### Secondary structure and interaction determinations by circular dichroism (CD)

To analyze the interaction among the molecules studied, equimolar combinations were prepared at 50 μM in PBS and co-incubated for 24h at 4°C. Incubation and measurement at low temperature limits aggregation and decreases the molecular dynamics characteristic of small peptides, both necessary to get an acceptable signal-to-noise ratio. Protein secondary structure was monitored at 4°C by far-UV CD spectroscopy from 260 nm to 190 nm in a Jasco J-715 spectrophotopolarimeter. Protein concentration was set to 50 μM, and 20 scans were recorded at 50 nm min^-1^ (response 1s) in a 0.1 cm pathlength cuvette. Comparison of spectra for the individual molecules at 50 μM and 100 μM indicated that they were not aggregated in the conditions used in this study.

### Secondary structure determination by Attenuated-Total Reflectance (ATR)-Fourier Transform InfraRed (FTIR) spectroscopy

Protein/peptides at 50μM in deuterated PBS were N_2_-dried onto an ATR-cell and 250 spectra were acquired at a resolution of 2 cm^-1^ and at room temperature in a Variant Resolutions Pro spectrometer.

### Transmission electron microscopy (TEM) analysis

To visualize the extent of aggregation and morphology of the different combinations, samples were diluted to 10μM in PBS and quickly adsorbed on to glow-discharge carbon-coated grids. Transmission electron microscopy was performed in a Jeol 120-kV JEM-1400 microscope, using 1% uranyl acetate for negative staining.

### Cell isolation and culture

Adult primary human astrocytes were isolated from brain specimens obtained at surgery (medication-refractory epilepsy) as described before [18,19]. The tissue had to be removed to reach the epileptic focus and was not needed for diagnostic purposes. Normal temporal cortex specimens from 5 patients were used (Table 1).

**Table 1:** Brain tissue donor characteristics. Demographics of patients who underwent surgery for therapy resistant epilepsy where normal. Neocortex was removed to reach the epileptic focus and was not needed for diagnostic purposes. Astrocytes isolated from these specimens were used for the Aβ uptake experiments.

ADULT HUMAN PRIMARY ASTROCYTE CULTURES				
CASE #	GENDER	AGE (YEARS)	APOE GENOTYPE	PASSAGE NUMBER
**1**	**Female**	**42**	**E2/E3**	**5**
**2**	**Male**	**19**	**E3/E4**	**4**
**3**	**Female**	**21**	**E3/E3**	**4**
**4**	**Female**	**53**	**E3/E3**	**3**
**5**	**Female**	**31**	**E3/E3**	**5**

Permission for the use of human brain tissue for *in vitro* research was granted by the Ethical Medical Committee of the VU University Medical Center (VUmc) in Amsterdam, where the operations took place. Brain tissue specimens were obtained with written informed consent and patient information was treated in accordance with the Declaration of Helsinki.

Isolated astrocytes were cultured in medium containing a mixture of DMEM and HAM-F10 (Gibco) (1:1) supplemented with 10%(v/v) fetal calf serum (Hycult), L-glutamin (2 mM; Gibco), penicillin(100 IU/mL) and streptomycin (50 g/mL) (both Gibco) at 37°C and 5% CO2[18]. Astrocytes were used up to passage 7.

### Cell treatment and flow cytometry

Astrocytes were plated at a density of 25–50,000 cells per well in a 24-well plate. Cells were allowed to adhere for 48h before exposure to different preincubation mixtures of scFv-h3D6/apoE-MP/apoJ-MP and Aβ. After 18h, cell culture supernatant was collected, centrifuged (300xg for 5 min) and stored at -20°C until further analysis. Cells were rinsed with PBS and 0.2% trypan blue was added (for 2 minutes) to quench the signal of extracellular Aβ oligomers and Aβ fibrils. Then, cells were rinsed again with PBS and harvested using 0.25% trypsin (w:v), centrifuged for 5 min at 300xg and washed three times in cold FACS buffer (0.25% BSA in PBS(w:v). Cellular Aβ-uptake of FAM-labeled Aβ_1–42_ was quantified using flow cytometry (FACS) as described before[18]. The percentage of Aβ positive astrocytes, 10,000 counted cells per condition, was quantified using the FACS Calibur (BD Biosciences, San Jose, CA) with the CellQuest software. Cells were gated based on morphological appearance (forward and side scatter) to count all viable cells and exclude cellular debris and dead cells.

### Statistics

Statistical analysis was performed using Graphpad 6. Differences among the various treatments were assessed with a paired t-test using raw data. All data were expressed as means±SEM values and a *P* value of <0.05 was considered to reflect statistical significance.

## Results

### Secondary structure of scFv-h3D6, Aβ, apoE-MP and apoJ-MP

**Fig 1:**
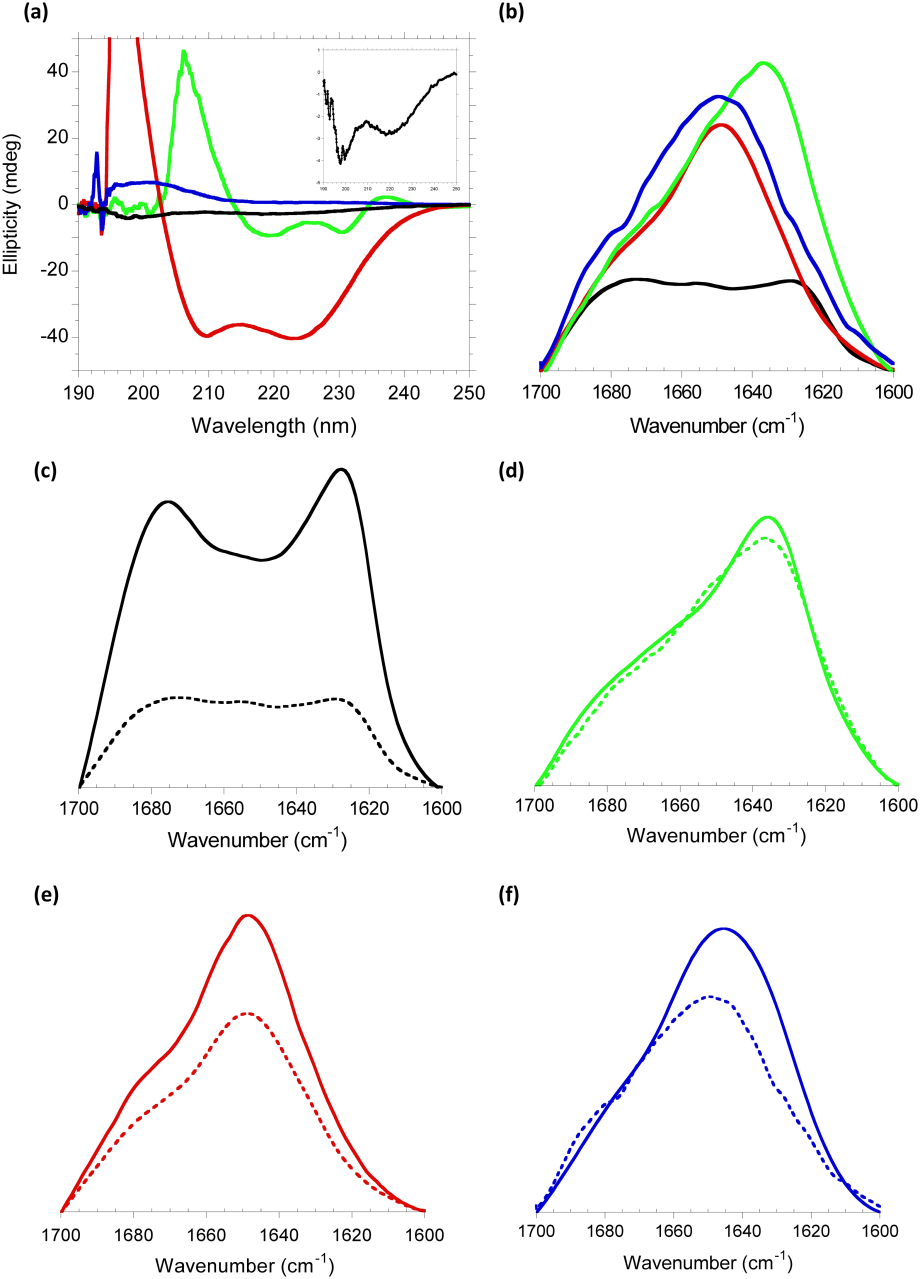
Secondary structure of the different molecules assayed. **(a)** Circular Dichroism and **(b)** ATR-FTIR spectra of Aβ peptide (black), scFv-h3D6 (green), apoE-MP (red) and apoJ-MP (blue) at 4°C. The inset in **(a)** shows the CD spectrum of the Aβ peptide in a smaller scale. **(c-f)** ATR-FTIR spectra of samples incubated at 4°C (dotted lines) and 37°C (continuous lines) of **(c)** Aβ, **(d)** scFv-h3D6, **(e)** apoE-MP and **(f)** apoJ-MP.

Characterization of the secondary structure of each of the molecules involved in this study is required to next identify interacting partners. CD was recorded at 4°C to decrease the molecular dynamics characteristic of small peptides, to get an acceptable signal-to-noise ratio, and to partially prevent aggregation of the Aβ peptide. ATR-FTIR allows the measurement of protein aggregates. Fig 1A shows far-UV CD spectra at 4°C for these molecules. ScFv-h3D6 spectrum displays the canonical signatures for an all-β protein, a minimum at 218 nm and a maximum at around 200 nm. In addition, the spectrum shows a minimum at 230 nm and a positive shoulder at 237 nm that have been previously assigned to the particular contribution of the Trp residues in the core of the variable domains to the far-UV region[40]. The Aβ peptide spectrum has a very low signal, but undoubtedly its shape is that of the combination of random coil (minimum at 198 nm) and β-sheet (minimum at 218 nm) conformations (see inset in Fig 1A)[41]. As expected from the NMR structure of the full-length apoE[42], the apoE-MP spectrum shows a pure α-helix conformation, with the typical minima at 222 nm and 208 nm. The apoJ-MP spectrum is singular, the signal is positive, because it was synthetized from D-aa to prevent proteolysis *in vivo* of this short and unstructured peptide. Therefore, the spectrum is the mirror-image of the expected for an L-isomer and corresponds to a random coil conformation. This concurs with a minimal helicity previously described for this peptide in the same conditions (13%)[32], which is reported to increase in the presence of lipids[43].

Fig 1B shows ATR-FTIR spectra in the amide I’ region for the different molecules studied. Samples were pre-incubated at either 4°C or 37°C to get oligomers or fibrils, respectively, before acquiring the spectra at room temperature. When pre-incubated at 4°C, the scFv-h3D6 spectrum is centered at 1638 cm^-1^, which is indicative of the prevalence of the native β-sheet component, as we previously described in depth[28,37]. The Aβ peptide spectrum is rather flat at this temperature, and assignment of its components is challenging. The apoE-MP spectrum is centered at 1650 cm^-1^, which fits with the prevalence of an α-helix component. The apoJ-MP spectrum is wider and could fit a random coil conformation. The spectra changed their shape depending on the temperature at which previous incubation was done in the case of Aβ (Fig 1C) and apoJ-MP (Fig 1F) but not for scFv-h3D6 nor apoE-MP (Fig 1D and 1E). This fact makes sense since the conformation of small or unstructured peptides is quite temperature-dependent. For Aβ, pre-incubation of the preparation at 37°C induced the appearance of two separate components characteristic of amyloid fibrils, located at around 1680 and 1620 cm^-1^. ApoJ-MP spectrum slightly shifted to lower frequencies, which could be indicative of some extent of aggregation at 37°C.

### Interactions among scFv-h3D6, Aβ, and apoE-MP or apoJ-MP

**Fig 2:**
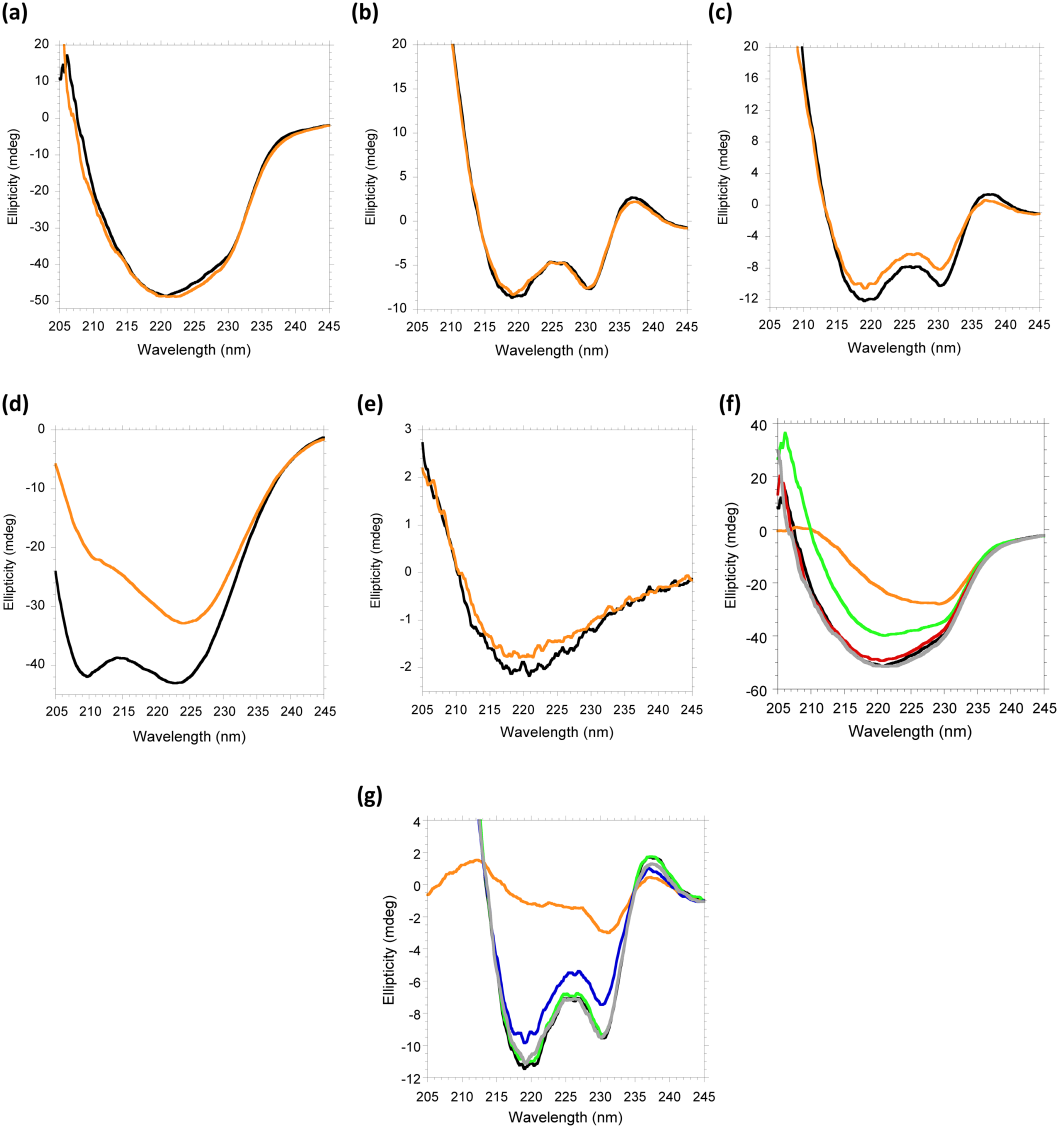
Interactions assessed by circular dichroism. Arithmetic sum of the spectra of each molecule alone (black) *vs* experimental spectra of different mixtures (orange). **(a)** scFv-h3D6+apoE-MP; **(b)** scFv-h3D6+apoJ-MP; **(c)** scFv-h3D6+Aβ; **(d)** apoE-MP+Aβ; **(e)** apoJ-MP+Aβ; the conformational change is evident for apoE-MP+Aβ (d); clear for scFv-h3D6+Aβ (c), and faint for apoJ-MP+Aβ (e). The sum of the experimental spectra for the different pairs assayed and the third component are compared in f-g. **(f)** Arithmetic sum scFv-h3D6+apoE-MP+Aβ, black; Experimental scFv-h3D6+apoE-MP+Aβ, orange; Experimental (scFv-h3D6+Aβ) + apoE-MP, red; Experimental (apoE-MP+Aβ) + scFv-h3D6, green; Experimental (apoE-MP+scFv-h3D6) + Aβ, grey. The sum of the experimental spectra for the apoE-MP+Aβ mixture and scFv-h3D6 (green) is the different one, indicating that apoE-MP sequesters Aβ and does not allow it to interact with scFv-h3D6. **(g)** Arithmetic sum scFv-h3D6+apoJ-MP+Aβ, black; Experimental scFv-h3D6+apoJ-MP+Aβ, orange; Experimental (scFv-h3D6+Aβ) + apoJ-MP, blue; Experimental (apoJ-MP+Aβ) + scFv-h3D6, green; Experimental (apoJ-MP+scFv-h3D6) + Aβ, grey line. The sum of the experimental spectra for the scFv-h3D6+Aβ mixture and apoJ-MP (blue) is the different one, indicating that the interaction scFv-h3D6/Aβ predominates.

One of the advantages of CD spectroscopy is that the arithmetic sum of two spectra may overlap the experimental spectrum of the equimolar mixture if there is no interaction. If interaction exists, the docking of both molecules may change their conformation so that the experimental and the arithmetic spectra would differ. It is important to note that the conformational change induced by binding can be too small to be detected by this methodology, and it does not correlate with the strength of the interaction. However, when a conformational change is detected by CD, the interaction unequivocally exits. On the other hand, differences between the experimental CD-spectrum and the arithmetic sum are indicative of aggregation of the complex if the experimental spectrum is similar in shape but less intense than the sum. Fig 2 shows the spectra for several combinations of scFv-h3D6, apoE-MP, apoJ-MP and Aβ assayed.

ScFv-h3D6 was not detected interacting with either apoE-MP or apoJ-MP (Fig 2A and 2B), but as expected it was with Aβ (Fig 2C). ApoE-MP also interacted with Aβ (Fig 2D). The conformational change was faint when mixing apoJ-MP and Aβ, plausibly due to the small size of the apoJ-MP (10 residues) and/or to a weak interaction (Fig 2E).

When combining scFv-h3D6, apoE-MP and Aβ, the obtained spectrum was different from the sum of the three individual spectra (Fig 2F). To hypothesize on the nature of the complex, we compared the sums of the experimental spectra for the different pairs assayed and the third component. Only the sum of the spectra for apoE-MP+Aβ and scFv-h3D6 differed from the other spectra (Fig 2F). This would indicate that apoE-MP sequesters Aβ and does not allow it to interact with scFv-h3D6, which may prevent the formation of protective WL fibrils (see below). When combining scFv-h3D6, apoJ-MP and Aβ, the obtained spectrum differs from the sum of the three individual spectra (Fig 2G). When comparing the sum of the experimental spectra for the different pairs and the third component, the differences are displayed by the sum of the spectra for scFv-h3D6+Aβ and apoJ-MP, which, at first glance, would indicate that the interaction scFv-h3D6/Aβ predominates. However, because the previous observation of the possibility that the small size of apoJ-MP does not induce a huge conformational change, makes other complementary information necessary (see below).

**Fig 3:**
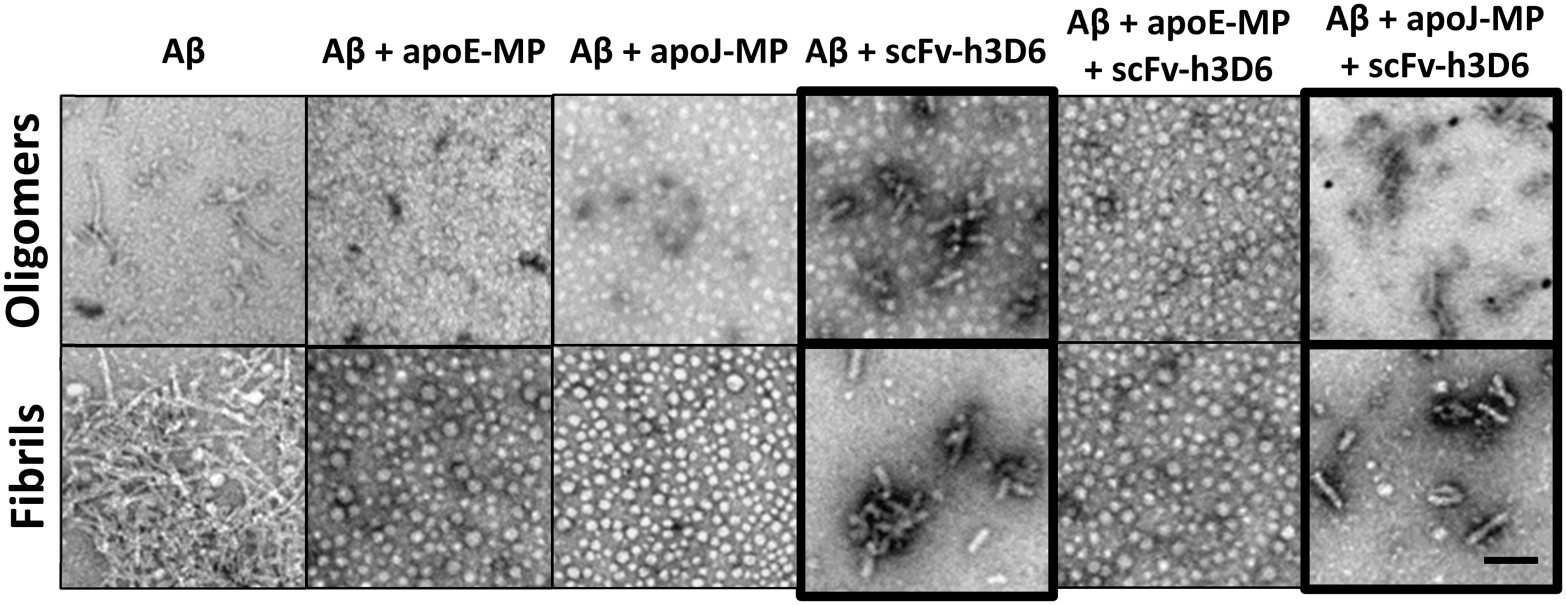
TEM micrographs. To visualize the extent of aggregation and the morphology of the different combinations, samples were diluted to 10μM in PBS and quickly adsorbed on to glow-discharge carbon-coated grids. Transmission electron microscopy was performed in a Jeol 120-kV JEM-1400 microscope, using 1% uranyl acetate for negative staining. Worm-like fibrils are outlined. Bar is 1μm.

As mentioned above, the scFv-h3D6/Aβ complex aggregates as WL fibrils and this is the molecular basis of its protective effect[28]. It is interesting to notice that the formation of WL fibrils is an intrinsic property of the scFv-h3D6, and that such an aggregation pathway is thermodynamically and kinetically favoured when the scFv-h3D6 and Aβ form a complex, explaining how the scFv-h3D6 withdraws Aβ oligomers from the amyloid pathway and, consequently, how cytotoxicity is avoided. Therefore, we aimed at assessing the formation of WL fibrils in preparations of Aβ oligomers or fibrils and all combinations of scFv-h3D6, apoJ-MP and apoE-MP by Transmission Electron Microscopy (TEM) (Fig 3). In the presence of apoE-MP and scFv-h3D6, Aβ oligomers had a globulomer-like appearance, supporting the CD experiments conclusion that apoE-MP sequesters Aβ and precludes the scFv-h3D6/Aβ interaction, and the subsequent formation of WL fibrils. ApoJ-MP induced a faint conformational change when binding to Aβ and no such a change was observed when mixing with scFv-h3D6. When the three molecules were together the interaction between scFv-h3D6 and Aβ predominated, as judged from the formation of WL fibrils (Fig 3 (oligomers)). The Aβ peptide alone clearly formed amyloid fibrils, when incubated according to the protocol for fibril formation. No amyloid fibrils could be observed (Fig 3 (fibrils)), when Aβ was allowed to form fibrils in the presence of apoE-MP or apoJ-MP. This means that both MPs bind Aβ. When scFv-h3D6 and Aβ were co-incubated, WL fibrils were formed, also in the presence of apoJ-MP, but not when apoE-MP was involved, as was seen with oligomers. Therefore, TEM results were the same for oligomers than for fibrils and concur with CD data: apoE-MP interferes WL fibril formation by binding the Aβ peptide whereas apoJ-MP does not. As a consequence, the faint conformational change detected by CD when mixing apoJ-MP and Aβ would be due to a weak interaction rather than to a small conformational change upon binding.

### Uptake of Aβ oligomers by astrocytes in the presence of scFv-h3D6 and apoE-MP or apoJ-MP

To assess the effect of scFv-h3D6, apoE-MP and apoJ-MP in Aβ oligomerization and fibrillation, and their consequences in Aβ uptake, the preparations of Aβ oligomers and Aβ fibrils were prepared as in the TEM experiments (Fig 3). Aβ was used at a 10 μM concentration, and apoE-MP and apoJ-MP at a concentration of 1 μM (ratio 10:1), found to be the most effective concentration to test full-length human apolipoproteins E and J in previous studies[19]. ScFv-h3D6 was tested in a range of different concentrations (0, 1, 5, and 10 μM), and a dose-dependent effect on Aβ uptake by adult human astrocytes was observed. One μM concentration of scFv-h3D6 was chosen for further experiments, because it reduced Aβ uptake to levels in which additional effects of the mimetic peptides could still be detected.

**Fig 4:**
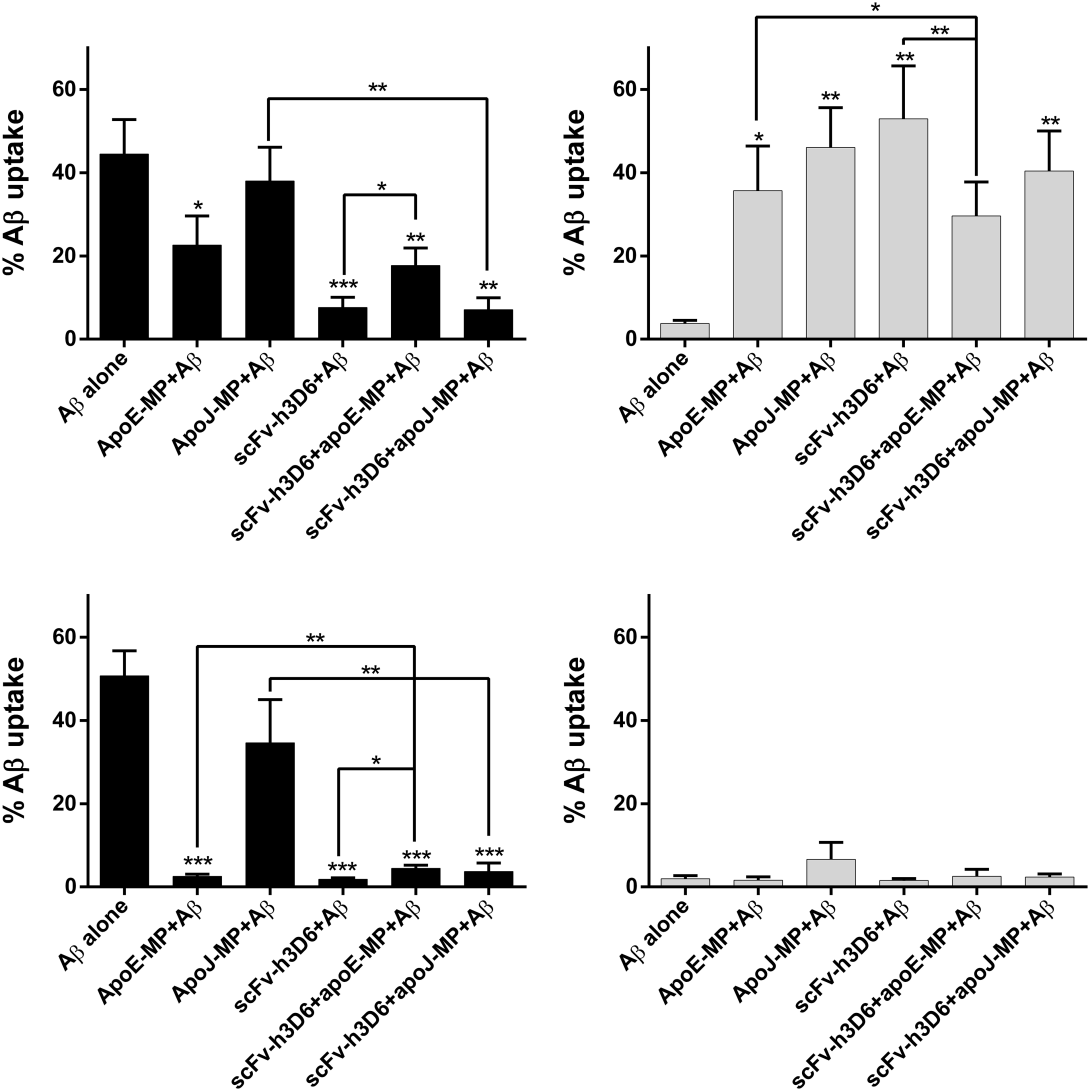
Effects of apoE-MP, apoJ-MP and scFv-h3D6 on Aβ oligomers or Aβ fibrils uptake by adult human astrocytes. Effects of apoE-MP, apoJ-MP and scFv-h3D6, either alone or in combination, on Aβ oligomers **(a, c)** or Aβ fibrils **(b, d)** uptake by astrocytes. Monomeric Aβ (100 μM) was allowed to aggregate into Aβ oligomers (24h at 4°C in phenol-red free DMEM) and Aβ fibrils (24h at 37°C in 10 mM HCl) in the absence and presence of the peptides and/or the antibody fragment. Aβ oligomers and Aβ fibrils were applied to the cells at a fixed concentration of 10 μM, as in previous studies, and scFv-h3D6, apoE-MP and apoJ-MP were tested on the cells at a 1 μM concentration **(a, b)**, or at a 2.5 μM concentration **(c, d)**. Results from a minimum of five individual astrocytes cultures from different patients are presented as means with standard error. *P<0.05, **P<0.01, ***P<0.001. Symbols above each column indicate significant changes due to the treatment compared to Aβ alone. Arrows show significance comparing two concrete treatments.

Five independent experiments were performed using human primary astrocytes from five different patients (Table 1). By the use of flow cytometry, we quantified the fraction of Aβ-positive astrocytes after 18h of exposure to either fluorescently labelled Aβ oligomers or fibrils, formed in either absence or presence of the different peptides and/or protein. Fig 4A shows how oligomeric Aβ uptake was reduced when Aβ was preincubated with either scFv-h3D6, apoE-MP or apoJ-MP. ScFv-h3D6 was shown to be more effective in reducing uptake (7.6%±2.3) than apoE-MP (22.6%±6.5) or apoJ-MP (38.0%±7.6), probably because it has higher affinity for Aβ than any of the apo-MPs.

When Aβ was preincubated to form oligomers in the presence of scFv-h3D6 and apoE-MP simultaneously, Aβ uptake was significantly increased (17.7%±3.9) compared to scFv-h3D6 alone (7.6%±2.3), suggesting that apoE-MP is precluding the interaction between scFv-h3D6 and Aβ oligomers, which makes scFv-h3D6 less effective in reducing Aβ uptake (Fig 4A). This concurs with the CD and TEM data above: (i) strong conformational change was induced by apoE-MP and Aβ interaction, and (ii) apoE-MP sequestered Aβ and did not allow it to interact with scFv-h3D6 and, consequently, protective WL fibrils were not observed.

ApoJ-MP did not affect the effect of scFv-h3D6 on uptake, (scFv-h3D6 alone (7.6%±2.3) and apoJ-MP+scFv-h3D6 (7.1%±2.7) (Fig 4A). This is consistent with the fact that apoJ-MP was not observed to interact with Aβ in the presence of scFv-h3D6, as inferred by CD and TEM. However, the addition of scFv-h3D6 significantly decreased uptake compared to apoJ-MP alone (38.0%±7.6). Thus, whereas apoE-MP interferes with the scFv-h3D6-induced reduction in Aβ oligomers uptake by astrocytes, apoJ-MP does not.

### Uptake of Aβ fibrils by astrocytes in the presence of scFv-h3D6 and apoE-MP or apoJ-MP

In a previous study, astrocytes were shown to bind and ingest Aβ oligomers more avidly than Aβ fibrils[19]. In the current study, we focused on ingested Aβ only, and used trypan blue for quenching the signal of the extracellular Aβ. This reduced the signal (% fluorescence positive astrocytes and mean fluorescence per cell) more pronounced for Aβ fibrils compared to oligomers, probably because fibrils are not easily taken up by astrocytes and remain attached extracellularly. Fig 4B shows that in the case of fibrils (Aβ alone) the percentage of Aβ positive astrocytes was 3.8%±0.7, rather low when compared to Aβ oligomers uptake (Aβ alone in Fig 4A: 44.5%±7.7).

When scFv-h3D6, apoE-MP and apoJ-MP were preincubated, alone or combined, with Aβ monomers to form fibrils, Aβ uptake was increased to much higher levels than uptake of Aβ fibrils alone (Fig 4B). Values of the % Aβ positive cells ranged from 30% to 50%, similar to uptake levels of Aβ oligomers alone. The biggest effect was observed for scFv-h3D6, followed by apoJ-MP, and last apoE-MP. Slightly different results were obtained with Aβ oligomers, where scFv-h3D6 again was most potent, but apoE-MP was more effective than apoJ-MP (Fig 4A). This means that apoE-MP more intensely affected the formation of oligomers than fibrils, whereas apoJ behaved opposite. Combinations of scFv-h3D6 and either apoE-MP or apoJ-MP did not enhance Aβ uptake to an equal extent as either factor alone incubated with Aβ fibril preparations. This could be indicative of some competition between these molecules to preclude Aβ fibrillation.

Whether the main species remaining after co-incubation were monomers, was investigated by increasing the concentration of scFv-h3D6, apoE-MP and apoJ-MP from 1 μM to 2.5 μM, and their effect on Aβ uptake and fibrillation was monitored. At 2.5 μM, scFv-h3D6 reduced Aβ oligomers uptake from 50.7%±5.9 to 1.8±0.4%, apoE-MP to 2.6±0.6%, and apoJ-MP to 34.6±10.4% (Fig 4C), effects more pronounced than those obtained with 1 μM. The effect of the combination of scFv-h3D6+apoJ-MP (3.7±2.0%) was not significantly different from that of scFv-h3D6 alone, corroborating the small effect of apoJ-MP on Aβ oligomers uptake. However, the combination of scFv-h3D6+apoE-MP (8.7±1.5%) increased the uptake with respect to the values obtained with each of the molecules alone. This again points to a competition phenomenon between scFv-h3D6 and apoE-MP for binding Aβ.

No effect of any of these molecules on Aβ fibrils uptake was observed, possibly because Aβ fibrils were hardly taken up (1.9±0.8% Aβ positive cells, when exposed to Aβ fibrils alone; Fig 4D). This suggests that at higher concentrations, scFv-h3D6, apoE-MP, and apoJ-MP, apart from interfering with Aβ fibrillation, also more effectively bind Aβ oligomers and reduce uptake. Therefore, what we are seeing is the interplay between two opposite phenomena: the interference in fibrillation, increasing the concentration of oligomers with respect to the Aβ fibrils alone, and the binding of such oligomers precluding uptake.

### LDH release as an indicative of cytotoxicity of the different treatments tested in this study

Astrocytes have been described not to be affected by Aβ toxicity although long-term exposure can impair their function and spread partially degraded Aβ, which then becomes toxic for neurons [26]. Cell culture supernatant was collected before flow cytometry analysis and LDH levels were measured after exposure to each treatment. LDH is a cytoplasmic enzyme released when cells die by necrosis, as usually triggered by external factors such as toxic chemical or traumatic physical events. None of the treatments induced any significant difference in LDH levels after 18h hours of incubation.

## Discussion

ScFv-h3D6 is an Aβ-directed antibody fragment, shown to have therapeutic efficacy in the 3xTg-AD mouse model[29]. Among other beneficences, one single intraperitoneally-administered dose was sufficient to recover the non-pathological levels of the main apolipoproteins in the brain, apoE and apoJ, whose concentrations are rather high in this mouse model[29]. Both apolipoproteins are involved in Aβ clearance[44,45], and we previously hypothesized that they could also be involved in the clearance of the scFv-h3D6/Aβ complex[29]. In this work, we have studied the interaction among scFv-h3D6, the Aβ peptide, and apoE-MP or apoJ-MP to next test the influence of scFv-h3D6 in combination with these MPs on Aβ aggregation and cellular uptake by primary human astrocytes.

The conformation of the molecules used in this study was characterized by CD and ATR-FTIR, and all of them were correctly folded. When the interactions among them were studied, scFv-h3D6 was not observed to interact with either apoE-MP or apoJ-MP. ApoE-MP clearly interacted with Aβ, but minor effects of apoJ-MP on conformation were observed. This may either have resulted from a very weak interaction between apoJ-MP and Aβ, or even if there was a strong interaction in terms of K_D_, minor effects, because the small size of the apoJ-MP is not likely to induce a huge conformational change upon binding. When mixing Aβ, scFv-h3D6 and apoJ-MP, scFv-h3D6/Aβ complexes and ultimately WL fibrils are formed, indicating that the interaction between Aβ and apoJ-MP is rather weak. On the contrary, apoE-MP certainly interacted with Aβ and precluded the formation of WL fibrils. Therefore, at first glance, the combination of scFv-h3D6 with apoE-MP might result in the loss of function of the scFv-h3D6, whereas with apoJ-MP the therapeutic potential remains.

These interactions were further indirectly analyzed by flow cytometry. ScFv-h3D6 was more efficient in reducing Aβ oligomers uptake than apoE-MP or apoJ-MP, as expected from the high affinity of an antibody fragment. In addition to this strong interaction, it is described that the scFv-h3D6/Aβ complex irreversibly aggregates in the form of WL fibrils and thus the trapped Aβ oligomers cannot be taken up. In the binding of the mimetic peptides to Aβ, apart from displaying lower affinities than scFv-h3D6, the reaction should present some degree of reversibility so that a fraction of Aβ oligomers could be dissociated from the complex and in that way become free for being taken up. It is already described that apoE significantly decrease uptake of Aβ by astrocytes[19,46], so that apoE-MP is emulating the function of the full-length apolipoprotein, at least in terms of astrocytic uptake. However, other beneficences of the full-length apoE are not likely to be displayed by the apoE-MP. It is known that apoE facilitates the proteolytic clearance of soluble Aβ from the brain, and that such a capacity is modulated by the apoE isoform and lipidation status [47]. Concretely, apoE dramatically enhances the endolytic degradation within microglia by neprilysin and the extracellular degradation by insulin-degrading enzyme. The potentiation of Aβ clearance by degradation, and the subsequent amelioration of AD hallmarks, has recently been demonstrated *in vivo* by inducing the transcription and lipidation of apoE through treatment with an agonist for the liver X receptors, in combination with the further induction of the transcription of apoE and the reduction in the microglial expression of proinflammatory genes through treatment with an agonist of the peroxisome-proliferator receptor γ [48].

When scFv-3D6 treatment was combined with apoE-MP, apoE-MP partially precluded the interaction between scFv-h3D6 and Aβ oligomers and therefore scFv-h3D6 was not so effective in reducing Aβ uptake. ApoJ-MP, which alone decreased Aβ oligomers uptake although not significantly, showed a value similar to scFv-h3D6 when both molecules were combined so apoJ-MP does not substantially affect Aβ oligomers uptake by astrocytes.

Aβ fibrils have been described not to be so easily taken up by astrocytes compared to Aβ oligomers [18,19]. Here, the uptake of Aβ fibrils was 10% that of Aβ oligomers. Strikingly, when Aβ monomers were incubated to form fibrils in the presence of scFv-h3D6, apoE-MP or apoJ-MP at 1 μM, Aβ uptake by astrocytes was increased. This implies that in the presence of any of these three molecules, Aβ monomers cannot aggregate to form fibrils probably due to the interaction of the molecules with low molecular weight Aβ aggregates, or even monomers. The fact that apoE and apoJ interfere with Aβ fibril formation explains why they are co-localized with Aβ amyloid plaques in AD brain[49]. In this sense, it has been suggested that apoJ can influence transthyretin fibril formation but may not modulate toxicity[50]. In AD, some authors have pointed out that apoJ facilitates amyloid formation[51], while others have shown that it inhibits Aβ fibrillation *in vitro* and promotes the clearance of protein aggregates via endocytosis[52]. ApoE has also been described to prevent Aβ fibrillogenesis[53], regardless its isoform[54,55], as Aβ is able to interact with both the lipid-binding site and the receptor-binding site within apoE[11]. Since the apoE-MP used in this work corresponds to the receptor binding site of apoE, equal in all human isoforms, and a similar Class A lipid associating domain[31], the binding to Aβ may be occurring, at least, through the receptor binding site. When combining scFv-h3D6 with apoE or apoJ-MP the increase in Aβ uptake was lower than with each of the three molecules alone, especially for the combination with apoE-MP. This would indicate that the mechanism by which scFv-h3D6 interferes with fibrillation is different from that by the peptides and that both mechanisms are somehow antagonistic.

When the concentration of scFv-h3D6 and MPs was increased from 1 to 2.5 μM, strikingly Aβ uptake was reduced to similar levels as Aβ fibril alone. Therefore, a higher concentration of scFv-h3D6, apoE-MP, and apoJ-MP, apart from better preventing Aβ fibrillation, could also more effectively reduce Aβ uptake. This makes sense, since the number of molecules is higher in a preparation of monomers than in a preparation of Aβ oligomers or Aβ fibrils, and so concentration has a great effect because it favors the binding of monomers and/or small-sized oligomers to scFv-h3D6, apoE-MP, and apoJ-MP.

Summarizing, scFv-h3D6, apoE-MP and apoJ-MP have been shown to prevent Aβ fibrillation and reduce Aβ uptake by human primary astrocytes. However, the combination of apoE-MP with scFv-h3D6 interfered with the dramatic reduction on Aβ uptake by the anti-Aβ antibody fragment and precluded the formation of protective WL fibrils, while apoJ-MP did not. So, none of these approaches, alone or combined, can be proposed to induce Aβ degradation and clearance intracellularly. However, apoE-MP and scFv-h3D6 have previously been described to improve Aβ pathology *in vivo* [29,31]. Apart from the impaired function generated by long-term exposure to an overload of Aβ inside the astrocytes [26,56], it has to be considered that glial cells lose their capability of degrading Aβ with aging and, in the case of AD, with the chronic neuroinflammation associated with the disease[57,58]. Both facts point to a negative effect when inducing Aβ ingestion as an approach to reduce Aβ overburden. Therefore, the traditional idea of designing therapeutic approaches to promote Aβ clearance by glial cells, like complete monoclonal antibodies, which are taken up by glial cells through Fc receptors [59], should be carefully re-evaluated. Deviation of Aβ clearance from a glial uptake pathway towards clearance through BBB or brain-CSF barriers has been shown to be effective [60,61] and may be more advantageous since it could complement, and at the same time relax, the apoE-induced endolytic degradation within microglia [46].

## Conclusions

As a general conclusion, apoE-MP precluded the formation of protective WL fibrils by the scFv-h3D6/Aβ complex and partially interfered with the dramatic scFv-h3D6-induced reduction in Aβ-uptake by astrocytes, whereas apoJ-MP allowed the formation of WL fibrils and did not interfere with the reduction in Aβ uptake. As sustained Aβ uptake may impair astrocyte normal functions and ultimately neuronal viability, this work shows another beneficence of scFv-h3D6 treatment, which is not further improved by the use of apoE or apoJ mimetic peptides.
